# *cis*-*N*-(2-Hydroxy­cyclo­hexyl)-*p*-toluene­sulfonamide

**DOI:** 10.1107/S1600536810002151

**Published:** 2010-01-27

**Authors:** Mohamed I. Fadlalla, Holger B. Friedrich, Glenn E. M. Maguire, Muhammad D. Bala

**Affiliations:** a School of Chemistry, University of KwaZulu-Natal, Westville Campus, Private Bag X54001, Durban 4000, South Africa

## Abstract

There are two symmetry-independent mol­ecules in the asymmetric unit of the title compound, C_13_H_19_NO_3_S. The cyclo­hexane rings in the two mol­ecules adopt chair configurations. The hydr­oxy and amino groups on the cyclo­hexane ring assume axial and equatorial orientations, respectively, with respect to the plane of the ring. The crystal structure is stabilized by two inter­molecular N—H⋯O and O—H⋯O hydrogen bonds from the two symmetry-independent mol­ecules.

## Related literature

For related structures of β-amino alcohols, see: Bergmeier (2000 ▶); Krzemiński & Wojtczak (2005 ▶). For related structures of tosyl­amino compounds, see: Coote *et al.* (2008 ▶); Liu *et al.* (2005 ▶); Chinnakali *et al.* (2007 ▶); Nan & Xing (2006 ▶). For the synthesis of the title compound, see: Naiker *et al.* (2008 ▶).
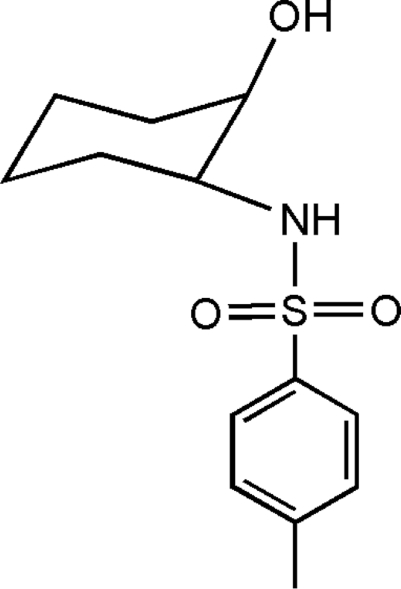


## Experimental

### 

#### Crystal data

C_13_H_19_NO_3_S
*M* *_r_* = 269.35
Triclinic, 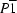

*a* = 6.3031 (1) Å
*b* = 12.8355 (2) Å
*c* = 17.5367 (3) Å
α = 106.645 (1)°
β = 93.971 (1)°
γ = 100.047 (1)°
*V* = 1327.75 (4) Å^3^
*Z* = 4
Mo *K*α radiation
μ = 0.24 mm^−1^
*T* = 173 K
0.51 × 0.31 × 0.25 mm

#### Data collection

Bruker APEXII CCD diffractometer
18458 measured reflections
6423 independent reflections
4837 reflections with *I* > 2σ(*I*)
*R*_int_ = 0.033

#### Refinement

*R*[*F* ^2^ > 2σ(*F* ^2^)] = 0.036
*wR*(*F* ^2^) = 0.111
*S* = 1.07
6423 reflections
343 parameters
H atoms treated by a mixture of independent and constrained refinement
Δρ_max_ = 0.40 e Å^−3^
Δρ_min_ = −0.41 e Å^−3^

### 

Data collection: *APEX2* (Bruker, 2005 ▶); cell refinement: *SAINT* (Bruker, 2005 ▶); data reduction: *SAINT*; program(s) used to solve structure: *SHELXS97* (Sheldrick, 2008 ▶); program(s) used to refine structure: *SHELXL97* (Sheldrick, 2008 ▶); molecular graphics: *ORTEP-3* (Farrugia, 1997 ▶) and *DIAMOND* (Brandenburg, 1998 ▶); software used to prepare material for publication: *SHELXTL* (Sheldrick, 2008 ▶).

## Supplementary Material

Crystal structure: contains datablocks global, I. DOI: 10.1107/S1600536810002151/lx2131sup1.cif

Structure factors: contains datablocks I. DOI: 10.1107/S1600536810002151/lx2131Isup2.hkl

Additional supplementary materials:  crystallographic information; 3D view; checkCIF report

## Figures and Tables

**Table 1:** Hydrogen-bond geometry (Å, °)

*D*—H⋯*A*	*D*—H	H⋯*A*	*D*⋯*A*	*D*—H⋯*A*
N1—H1*N*⋯O6^i^	0.83 (2)	2.00 (2)	2.8255 (17)	175.0 (18)
N2—H2*N*⋯O3^ii^	0.82 (2)	2.00 (2)	2.8155 (18)	173.1 (19)
O3—H3*O*⋯O5^iii^	0.83 (2)	1.93 (2)	2.7489 (15)	171 (2)
O6—H6*O*⋯O2^iv^	0.83 (2)	1.98 (2)	2.8001 (15)	169 (2)

Symmetry codes: (i) 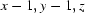; (ii) 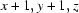; (iii) 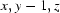; (iv) 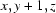.

**Fig. 1.**
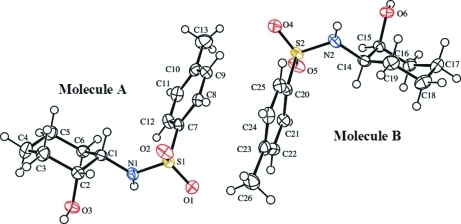
The molecular structure of the title compound with the atom numbering scheme. Displacement ellipsoids are drawn at the 50 % probability level. H atoms are presented as a small spheres of arbitrary radius.

**Fig. 2.**
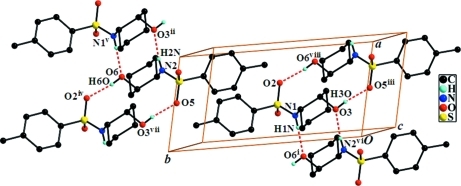
N—H···O and O—H···O hydrogen bonding interactions (dotted lines) in the crystal structure of the title compound. [Symmetry codes: (i) x - 1, y - 1, z; (ii) x + 1, y + 1, z; (iii) x, y - 1, z; (iv) x, y + 1, z; (v) x + 1, y + 1, z; (vi) x - 1, y - 1, z; (vii) x, y + 1, z; (viii) x, y - 1, z.]

## supplementary crystallographic information

### Comment

Molecules containing a β-amino alcohol system have been used as precursors for the synthesis of chiral ligands, aziridine and biologically active compounds (Bergmeier, 2000; Krzemiński & Wojtczak, 2005). As a part of study on this family of compounds, we report the crystal structure of the title compound (l) (Fig. 1).

The geometry of the benzenesulfonamide unit in (I) agrees with that for related structures (Chinnakali *et al.* 2007; Nan & Xing, 2006). The cyclohexane rings in the two molecules adopt the chair configuration. The hydroxy and amino groups on the cyclohexane ring respectively assume axial and equatorial orientations with respect to the plane of the ring. The crystal packing (Fig. 2) is stabilized by intermolecular N—H···O and O—H···O hydrogen bonds from the two neighbouring symmetry-independent molecules (Table 1).

### Experimental

The synthesis of the title compound was carried out using a modified literature method (Naiker *et al.* 2008) using a catalytic process. To a nitrogen saturated Schlenk tube, toluene (6 ml), water (172 µl) chloroamine-T (0.21 g, 0.956 mmol), cyclohexene (0.478 mmol) and catalyst (0.03 g) were added in that order. After the complete conversion of the starting material the catalyst was gravity filtered. The reaction mixture was washed with 15 ml of sodium sulfite (1 g in 15 ml of de-ionized water), followed by 15 ml of ethyl acetate. Then the aqueous layer was separated from the organic layer and washed further with 3 × 15 ml of ethyl acetate. The solvent was removed *in vacuo*, and the crude product was purified using preparative high pressure liquid chromatography to yield the title compound as a white solid. Single crystals suitable for X-ray diffraction were prepared by evaporation of a solution of the title compound in acetonitrile/water (1:1 v/v) at room temperature. (mp; 414–416 K) Spectroscopic analysis: ^13^C NMR (400 MHz, CDCl_3_, δ, p.p.m): = 19.76 (s, 1 C), 21.54 (s, 2 C), 27.98 (s, 1 C), 31.46(s, 1 C), 55.10 (s, 1 C), 68.76 (s, 1 C), 126.97 (s, 2 C), 129.74 (s, 2 C), 137.98(s, 1 C), 143.39 (s, 143.39).. MS m/*z* –[fragment]–(%): 291.8 (*M* + Na^+^) calculated = 291.8 for C_13_H_19_NO_3_SNa^+^.

FT–IR (cm^-1^): = 3414(*m*), (OH), 3137(*m*), (NH), 2938(w), 2849(w), 1598(*m*), (ar), 1059(*m*), (S=O).

### Refinement

All H-atoms were refined using a riding model, with C—H = 0.93 Å and *U*_iso_(H) = 1.2*U*_eq_(C) for aromatic, C—H = 0.97 Å and *U*_iso_(H) = 1.2*U*_eq_(C) for CH_2_, C—H = 0.96 Å and *U*_iso_(H) = 1.5*U*_eq_(C) for CH_3_, N—H = 0.86 Å and *U*_iso_(H) = 1.2*U*_eq_(N) for NH, and O—H = 0.82 Å and *U*_iso_(H) = 1.5*U*_eq_(O) for OH.

### Crystal data

**Table:**

C_13_H_19_NO_3_S	*Z* = 4
*M**_r_* = 269.35	*F*(000) = 576
Triclinic, *P*1	*D*_x_ = 1.347 Mg m^−^^3^
Hall symbol: -P 1	Melting point = 414–416 K
*a* = 6.3031 (1) Å	Mo *K*α radiation, λ = 0.71073 Å
*b* = 12.8355 (2) Å	Cell parameters from 6946 reflections
*c* = 17.5367 (3) Å	θ = 2.4–28.3°
α = 106.645 (1)°	µ = 0.24 mm^−^^1^
β = 93.971 (1)°	*T* = 173 K
γ = 100.047 (1)°	Block, colourless
*V* = 1327.75 (4) Å^3^	0.51 × 0.31 × 0.25 mm

### Data collection

**Table:**

Bruker APEXII CCD diffractometer	4837 reflections with *I* > 2σ(*I*)
Radiation source: fine-focus sealed tube	*R*_int_ = 0.033
graphite	θ_max_ = 28.0°, θ_min_ = 1.2°
φ and ω scans	*h* = −8→8
18458 measured reflections	*k* = −16→16
6423 independent reflections	*l* = −23→23

### Refinement

**Table:**

Refinement on *F*^2^	Primary atom site location: structure-invariant direct methods
Least-squares matrix: full	Secondary atom site location: difference Fourier map
*R*[*F*^2^ > 2σ(*F*^2^)] = 0.036	Hydrogen site location: difference Fourier map
*wR*(*F*^2^) = 0.111	H atoms treated by a mixture of independent and constrained refinement
*S* = 1.07	*w* = 1/[σ^2^(*F*_o_^2^) + (0.0582*P*)^2^ + 0.0739*P*] where *P* = (*F*_o_^2^ + 2*F*_c_^2^)/3
6423 reflections	(Δ/σ)_max_ < 0.001
343 parameters	Δρ_max_ = 0.40 e Å^−^^3^
0 restraints	Δρ_min_ = −0.41 e Å^−^^3^

### Special details

**Table:**

Geometry. All e.s.d.'s (except the e.s.d. in the dihedral angle between two l.s. planes) are estimated using the full covariance matrix. The cell e.s.d.'s are taken into account individually in the estimation of e.s.d.'s in distances, angles and torsion angles; correlations between e.s.d.'s in cell parameters are only used when they are defined by crystal symmetry. An approximate (isotropic) treatment of cell e.s.d.'s is used for estimating e.s.d.'s involving l.s. planes.
Refinement. Refinement of *F*^2^ against ALL reflections. The weighted *R*-factor *wR* and goodness of fit *S* are based on *F*^2^, conventional *R*-factors *R* are based on *F*, with *F* set to zero for negative *F*^2^. The threshold expression of *F*^2^ > σ(*F*^2^) is used only for calculating *R*-factors(gt) *etc*. and is not relevant to the choice of reflections for refinement. *R*-factors based on *F*^2^ are statistically about twice as large as those based on *F*, and *R*- factors based on ALL data will be even larger.

### Fractional atomic coordinates and isotropic or equivalent isotropic displacement parameters (Å^2^)

**Table:**

	*x*	*y*	*z*	*U*_iso_*/*U*_eq_	
C1	0.3149 (2)	0.39725 (12)	0.35318 (8)	0.0230 (3)	
H1	0.4155	0.4675	0.3860	0.028*	
C2	0.4327 (2)	0.30139 (12)	0.34741 (9)	0.0227 (3)	
H2	0.5706	0.3156	0.3236	0.027*	
C3	0.4842 (3)	0.28788 (14)	0.42986 (9)	0.0303 (4)	
H3A	0.5896	0.3543	0.4639	0.036*	
H3B	0.5522	0.2225	0.4240	0.036*	
C4	0.2795 (3)	0.27272 (15)	0.47095 (10)	0.0344 (4)	
H4A	0.1800	0.2022	0.4399	0.041*	
H4B	0.3195	0.2684	0.5254	0.041*	
C5	0.1644 (3)	0.36911 (14)	0.47692 (9)	0.0337 (4)	
H5A	0.2583	0.4384	0.5127	0.040*	
H5B	0.0285	0.3557	0.5009	0.040*	
C6	0.1107 (3)	0.38351 (13)	0.39456 (9)	0.0266 (3)	
H6A	0.0034	0.3178	0.3607	0.032*	
H6B	0.0449	0.4497	0.4010	0.032*	
C7	0.3445 (2)	0.63056 (12)	0.29981 (8)	0.0212 (3)	
C8	0.5061 (2)	0.72537 (12)	0.31511 (9)	0.0238 (3)	
H8	0.6421	0.7204	0.2956	0.029*	
C9	0.4672 (3)	0.82718 (12)	0.35906 (9)	0.0282 (3)	
H9	0.5775	0.8920	0.3693	0.034*	
C10	0.2692 (3)	0.83612 (13)	0.38840 (9)	0.0290 (4)	
C11	0.1103 (3)	0.74045 (14)	0.37164 (10)	0.0310 (4)	
H11	−0.0257	0.7454	0.3911	0.037*	
C12	0.1439 (3)	0.63787 (13)	0.32735 (9)	0.0284 (3)	
H12	0.0320	0.5735	0.3159	0.034*	
C13	0.2270 (4)	0.94682 (16)	0.43611 (11)	0.0459 (5)	
H13A	0.0900	0.9582	0.4130	0.069*	
H13B	0.3460	1.0061	0.4345	0.069*	
H13C	0.2173	0.9480	0.4919	0.069*	
N1	0.2596 (2)	0.40722 (10)	0.27298 (8)	0.0243 (3)	
O1	0.30664 (19)	0.48034 (9)	0.15979 (6)	0.0319 (3)	
O2	0.62179 (17)	0.50634 (9)	0.26023 (7)	0.0322 (3)	
O3	0.29042 (18)	0.20373 (9)	0.29512 (6)	0.0262 (2)	
S1	0.39239 (6)	0.50145 (3)	0.24179 (2)	0.02302 (10)	
H1N	0.134 (3)	0.3817 (15)	0.2496 (11)	0.039 (5)*	
H3O	0.364 (3)	0.1556 (18)	0.2813 (12)	0.050 (6)*	
C14	0.7984 (3)	1.13220 (12)	0.14293 (9)	0.0260 (3)	
H14	0.6960	1.0621	0.1109	0.031*	
C15	0.6821 (2)	1.22875 (12)	0.14932 (8)	0.0221 (3)	
H15	0.5435	1.2142	0.1726	0.026*	
C16	0.6321 (3)	1.24306 (13)	0.06696 (9)	0.0275 (3)	
H16A	0.5649	1.3087	0.0729	0.033*	
H16B	0.5267	1.1769	0.0325	0.033*	
C17	0.8384 (3)	1.25814 (14)	0.02681 (9)	0.0311 (4)	
H17A	0.8013	1.2657	−0.0269	0.037*	
H17B	0.9405	1.3268	0.0594	0.037*	
C18	0.9460 (3)	1.15881 (15)	0.01851 (10)	0.0382 (4)	
H18A	1.0803	1.1699	−0.0068	0.046*	
H18B	0.8468	1.0909	−0.0167	0.046*	
C19	1.0017 (3)	1.14389 (14)	0.10043 (10)	0.0322 (4)	
H19A	1.1115	1.2088	0.1339	0.039*	
H19B	1.0647	1.0768	0.0934	0.039*	
C20	0.7614 (2)	0.89890 (11)	0.20252 (8)	0.0214 (3)	
C21	0.5911 (3)	0.82342 (12)	0.15054 (9)	0.0253 (3)	
H21	0.4570	0.8445	0.1409	0.030*	
C22	0.6186 (3)	0.71642 (13)	0.11252 (9)	0.0290 (3)	
H22	0.5021	0.6643	0.0769	0.035*	
C23	0.8145 (3)	0.68457 (12)	0.12593 (9)	0.0276 (3)	
C24	0.9834 (3)	0.76236 (13)	0.17751 (9)	0.0276 (3)	
H24	1.1182	0.7418	0.1867	0.033*	
C25	0.9594 (3)	0.86956 (13)	0.21587 (9)	0.0260 (3)	
H25	1.0767	0.9221	0.2508	0.031*	
C26	0.8384 (3)	0.56743 (14)	0.08535 (11)	0.0414 (4)	
H26A	0.7230	0.5151	0.0976	0.062*	
H26B	0.9802	0.5569	0.1048	0.062*	
H26C	0.8275	0.5541	0.0272	0.062*	
N2	0.8567 (2)	1.12308 (11)	0.22299 (8)	0.0285 (3)	
O4	0.81390 (19)	1.05591 (9)	0.33850 (6)	0.0326 (3)	
O5	0.49416 (18)	1.02921 (9)	0.23866 (7)	0.0345 (3)	
O6	0.82296 (18)	1.32622 (9)	0.20231 (6)	0.0246 (2)	
S2	0.72262 (6)	1.03181 (3)	0.25663 (2)	0.02444 (11)	
H2N	0.981 (3)	1.1520 (16)	0.2453 (11)	0.040 (6)*	
H6O	0.751 (4)	1.3754 (19)	0.2141 (13)	0.061 (7)*	

### Atomic displacement parameters (Å^2^)

**Table:**

	*U*^11^	*U*^22^	*U*^33^	*U*^12^	*U*^13^	*U*^23^
C1	0.0245 (8)	0.0189 (7)	0.0237 (7)	0.0036 (6)	−0.0012 (6)	0.0050 (6)
C2	0.0177 (7)	0.0233 (7)	0.0270 (8)	0.0037 (6)	0.0014 (6)	0.0080 (6)
C3	0.0286 (9)	0.0345 (9)	0.0307 (8)	0.0111 (7)	−0.0005 (7)	0.0128 (7)
C4	0.0389 (10)	0.0442 (10)	0.0277 (8)	0.0158 (8)	0.0068 (7)	0.0177 (7)
C5	0.0375 (10)	0.0382 (9)	0.0257 (8)	0.0127 (8)	0.0057 (7)	0.0068 (7)
C6	0.0293 (9)	0.0256 (8)	0.0260 (8)	0.0117 (7)	0.0049 (6)	0.0054 (6)
C7	0.0221 (8)	0.0212 (7)	0.0237 (7)	0.0071 (6)	0.0046 (6)	0.0099 (6)
C8	0.0237 (8)	0.0256 (8)	0.0240 (7)	0.0051 (6)	0.0049 (6)	0.0099 (6)
C9	0.0337 (9)	0.0227 (8)	0.0275 (8)	0.0039 (7)	0.0026 (7)	0.0080 (6)
C10	0.0394 (10)	0.0282 (8)	0.0226 (8)	0.0152 (7)	0.0027 (7)	0.0080 (6)
C11	0.0286 (9)	0.0383 (9)	0.0344 (9)	0.0170 (7)	0.0114 (7)	0.0164 (7)
C12	0.0235 (8)	0.0280 (8)	0.0382 (9)	0.0074 (6)	0.0069 (7)	0.0153 (7)
C13	0.0564 (13)	0.0389 (10)	0.0413 (11)	0.0240 (9)	0.0051 (9)	0.0021 (8)
N1	0.0210 (7)	0.0224 (6)	0.0285 (7)	−0.0005 (5)	−0.0031 (5)	0.0106 (5)
O1	0.0401 (7)	0.0300 (6)	0.0254 (6)	0.0060 (5)	0.0061 (5)	0.0087 (5)
O2	0.0210 (6)	0.0244 (6)	0.0502 (7)	0.0075 (5)	0.0079 (5)	0.0073 (5)
O3	0.0234 (6)	0.0204 (5)	0.0322 (6)	0.0070 (5)	0.0023 (5)	0.0026 (4)
S1	0.0216 (2)	0.01999 (19)	0.0284 (2)	0.00532 (14)	0.00508 (15)	0.00776 (15)
C14	0.0291 (8)	0.0184 (7)	0.0274 (8)	0.0021 (6)	−0.0020 (6)	0.0051 (6)
C15	0.0173 (7)	0.0232 (7)	0.0245 (7)	0.0023 (6)	0.0009 (6)	0.0069 (6)
C16	0.0258 (8)	0.0306 (8)	0.0256 (8)	0.0063 (7)	−0.0013 (6)	0.0084 (6)
C17	0.0329 (9)	0.0383 (9)	0.0236 (8)	0.0084 (7)	0.0055 (6)	0.0106 (7)
C18	0.0422 (11)	0.0415 (10)	0.0282 (9)	0.0140 (8)	0.0095 (8)	0.0022 (7)
C19	0.0356 (10)	0.0278 (8)	0.0343 (9)	0.0167 (7)	0.0075 (7)	0.0045 (7)
C20	0.0232 (8)	0.0187 (7)	0.0243 (7)	0.0055 (6)	0.0056 (6)	0.0085 (6)
C21	0.0230 (8)	0.0252 (8)	0.0289 (8)	0.0071 (6)	0.0026 (6)	0.0090 (6)
C22	0.0328 (9)	0.0240 (8)	0.0274 (8)	0.0046 (7)	0.0011 (7)	0.0048 (6)
C23	0.0380 (9)	0.0250 (8)	0.0261 (8)	0.0130 (7)	0.0150 (7)	0.0112 (6)
C24	0.0256 (8)	0.0324 (8)	0.0333 (8)	0.0140 (7)	0.0107 (6)	0.0167 (7)
C25	0.0219 (8)	0.0279 (8)	0.0302 (8)	0.0050 (6)	0.0033 (6)	0.0119 (6)
C26	0.0559 (13)	0.0276 (9)	0.0450 (11)	0.0177 (8)	0.0194 (9)	0.0092 (8)
N2	0.0258 (8)	0.0223 (7)	0.0366 (8)	−0.0015 (6)	−0.0052 (6)	0.0133 (6)
O4	0.0412 (7)	0.0254 (6)	0.0288 (6)	0.0038 (5)	0.0057 (5)	0.0059 (5)
O5	0.0235 (6)	0.0230 (6)	0.0539 (8)	0.0081 (5)	0.0060 (5)	0.0047 (5)
O6	0.0234 (6)	0.0209 (5)	0.0268 (6)	0.0066 (5)	0.0010 (4)	0.0023 (4)
S2	0.0242 (2)	0.01775 (18)	0.0308 (2)	0.00465 (15)	0.00403 (15)	0.00618 (15)

### Geometric parameters (Å, °)

**Table:**

C1—N1	1.472 (2)	C14—N2	1.4690 (19)
C1—C2	1.528 (2)	C14—C15	1.528 (2)
C1—C6	1.529 (2)	C14—C19	1.532 (2)
C1—H1	1.0000	C14—H14	1.0000
C2—O3	1.4325 (17)	C15—O6	1.4316 (17)
C2—C3	1.525 (2)	C15—C16	1.526 (2)
C2—H2	1.0000	C15—H15	1.0000
C3—C4	1.531 (2)	C16—C17	1.530 (2)
C3—H3A	0.9900	C16—H16A	0.9900
C3—H3B	0.9900	C16—H16B	0.9900
C4—C5	1.522 (2)	C17—C18	1.522 (2)
C4—H4A	0.9900	C17—H17A	0.9900
C4—H4B	0.9900	C17—H17B	0.9900
C5—C6	1.530 (2)	C18—C19	1.528 (2)
C5—H5A	0.9900	C18—H18A	0.9900
C5—H5B	0.9900	C18—H18B	0.9900
C6—H6A	0.9900	C19—H19A	0.9900
C6—H6B	0.9900	C19—H19B	0.9900
C7—C8	1.390 (2)	C20—C21	1.385 (2)
C7—C12	1.393 (2)	C20—C25	1.389 (2)
C7—S1	1.7674 (14)	C20—S2	1.7665 (14)
C8—C9	1.387 (2)	C21—C22	1.391 (2)
C8—H8	0.9500	C21—H21	0.9500
C9—C10	1.393 (2)	C22—C23	1.392 (2)
C9—H9	0.9500	C22—H22	0.9500
C10—C11	1.386 (2)	C23—C24	1.388 (2)
C10—C13	1.507 (2)	C23—C26	1.507 (2)
C11—C12	1.384 (2)	C24—C25	1.387 (2)
C11—H11	0.9500	C24—H24	0.9500
C12—H12	0.9500	C25—H25	0.9500
C13—H13A	0.9800	C26—H26A	0.9800
C13—H13B	0.9800	C26—H26B	0.9800
C13—H13C	0.9800	C26—H26C	0.9800
N1—S1	1.5975 (13)	N2—S2	1.5982 (13)
N1—H1N	0.83 (2)	N2—H2N	0.82 (2)
O1—S1	1.4322 (11)	O4—S2	1.4343 (12)
O2—S1	1.4461 (11)	O5—S2	1.4452 (12)
O3—H3O	0.83 (2)	O6—H6O	0.83 (2)
			
N1—C1—C2	110.49 (11)	N2—C14—C15	110.28 (12)
N1—C1—C6	110.35 (12)	N2—C14—C19	109.93 (13)
C2—C1—C6	111.65 (12)	C15—C14—C19	112.15 (12)
N1—C1—H1	108.1	N2—C14—H14	108.1
C2—C1—H1	108.1	C15—C14—H14	108.1
C6—C1—H1	108.1	C19—C14—H14	108.1
O3—C2—C3	110.68 (12)	O6—C15—C16	110.75 (12)
O3—C2—C1	106.45 (11)	O6—C15—C14	107.06 (11)
C3—C2—C1	110.94 (12)	C16—C15—C14	110.71 (12)
O3—C2—H2	109.6	O6—C15—H15	109.4
C3—C2—H2	109.6	C16—C15—H15	109.4
C1—C2—H2	109.6	C14—C15—H15	109.4
C2—C3—C4	111.59 (13)	C15—C16—C17	111.19 (13)
C2—C3—H3A	109.3	C15—C16—H16A	109.4
C4—C3—H3A	109.3	C17—C16—H16A	109.4
C2—C3—H3B	109.3	C15—C16—H16B	109.4
C4—C3—H3B	109.3	C17—C16—H16B	109.4
H3A—C3—H3B	108.0	H16A—C16—H16B	108.0
C5—C4—C3	110.62 (14)	C18—C17—C16	110.11 (14)
C5—C4—H4A	109.5	C18—C17—H17A	109.6
C3—C4—H4A	109.5	C16—C17—H17A	109.6
C5—C4—H4B	109.5	C18—C17—H17B	109.6
C3—C4—H4B	109.5	C16—C17—H17B	109.6
H4A—C4—H4B	108.1	H17A—C17—H17B	108.2
C4—C5—C6	111.54 (13)	C17—C18—C19	110.83 (13)
C4—C5—H5A	109.3	C17—C18—H18A	109.5
C6—C5—H5A	109.3	C19—C18—H18A	109.5
C4—C5—H5B	109.3	C17—C18—H18B	109.5
C6—C5—H5B	109.3	C19—C18—H18B	109.5
H5A—C5—H5B	108.0	H18A—C18—H18B	108.1
C1—C6—C5	110.98 (13)	C18—C19—C14	110.71 (14)
C1—C6—H6A	109.4	C18—C19—H19A	109.5
C5—C6—H6A	109.4	C14—C19—H19A	109.5
C1—C6—H6B	109.4	C18—C19—H19B	109.5
C5—C6—H6B	109.4	C14—C19—H19B	109.5
H6A—C6—H6B	108.0	H19A—C19—H19B	108.1
C8—C7—C12	120.34 (14)	C21—C20—C25	120.81 (14)
C8—C7—S1	119.33 (11)	C21—C20—S2	119.75 (11)
C12—C7—S1	120.29 (11)	C25—C20—S2	119.36 (11)
C9—C8—C7	119.46 (14)	C20—C21—C22	119.29 (14)
C9—C8—H8	120.3	C20—C21—H21	120.4
C7—C8—H8	120.3	C22—C21—H21	120.4
C8—C9—C10	121.16 (15)	C21—C22—C23	120.93 (15)
C8—C9—H9	119.4	C21—C22—H22	119.5
C10—C9—H9	119.4	C23—C22—H22	119.5
C11—C10—C9	118.17 (14)	C24—C23—C22	118.55 (14)
C11—C10—C13	120.69 (16)	C24—C23—C26	121.60 (15)
C9—C10—C13	121.13 (16)	C22—C23—C26	119.84 (16)
C12—C11—C10	121.93 (15)	C25—C24—C23	121.41 (14)
C12—C11—H11	119.0	C25—C24—H24	119.3
C10—C11—H11	119.0	C23—C24—H24	119.3
C11—C12—C7	118.92 (15)	C24—C25—C20	119.00 (14)
C11—C12—H12	120.5	C24—C25—H25	120.5
C7—C12—H12	120.5	C20—C25—H25	120.5
C10—C13—H13A	109.5	C23—C26—H26A	109.5
C10—C13—H13B	109.5	C23—C26—H26B	109.5
H13A—C13—H13B	109.5	H26A—C26—H26B	109.5
C10—C13—H13C	109.5	C23—C26—H26C	109.5
H13A—C13—H13C	109.5	H26A—C26—H26C	109.5
H13B—C13—H13C	109.5	H26B—C26—H26C	109.5
C1—N1—S1	122.50 (10)	C14—N2—S2	122.76 (11)
C1—N1—H1N	119.7 (13)	C14—N2—H2N	117.8 (13)
S1—N1—H1N	113.8 (13)	S2—N2—H2N	116.7 (13)
C2—O3—H3O	107.3 (15)	C15—O6—H6O	107.3 (16)
O1—S1—O2	118.31 (7)	O4—S2—O5	119.25 (7)
O1—S1—N1	107.26 (7)	O4—S2—N2	106.51 (7)
O2—S1—N1	108.38 (7)	O5—S2—N2	107.95 (7)
O1—S1—C7	109.32 (7)	O4—S2—C20	108.22 (7)
O2—S1—C7	105.55 (7)	O5—S2—C20	105.34 (7)
N1—S1—C7	107.60 (7)	N2—S2—C20	109.34 (7)
			
N1—C1—C2—O3	−57.65 (14)	N2—C14—C15—O6	56.00 (15)
C6—C1—C2—O3	65.57 (15)	C19—C14—C15—O6	−66.87 (15)
N1—C1—C2—C3	−178.14 (12)	N2—C14—C15—C16	176.81 (12)
C6—C1—C2—C3	−54.92 (16)	C19—C14—C15—C16	53.94 (16)
O3—C2—C3—C4	−62.41 (17)	O6—C15—C16—C17	62.91 (16)
C1—C2—C3—C4	55.53 (17)	C14—C15—C16—C17	−55.68 (16)
C2—C3—C4—C5	−56.05 (19)	C15—C16—C17—C18	58.13 (18)
C3—C4—C5—C6	55.88 (19)	C16—C17—C18—C19	−58.33 (19)
N1—C1—C6—C5	178.09 (12)	C17—C18—C19—C14	56.40 (19)
C2—C1—C6—C5	54.80 (16)	N2—C14—C19—C18	−177.42 (13)
C4—C5—C6—C1	−55.44 (18)	C15—C14—C19—C18	−54.35 (17)
C12—C7—C8—C9	0.9 (2)	C25—C20—C21—C22	1.2 (2)
S1—C7—C8—C9	178.53 (11)	S2—C20—C21—C22	−175.50 (11)
C7—C8—C9—C10	0.2 (2)	C20—C21—C22—C23	−0.1 (2)
C8—C9—C10—C11	−0.8 (2)	C21—C22—C23—C24	−0.7 (2)
C8—C9—C10—C13	−179.95 (15)	C21—C22—C23—C26	178.69 (14)
C9—C10—C11—C12	0.2 (2)	C22—C23—C24—C25	0.6 (2)
C13—C10—C11—C12	179.39 (15)	C26—C23—C24—C25	−178.82 (14)
C10—C11—C12—C7	0.9 (2)	C23—C24—C25—C20	0.4 (2)
C8—C7—C12—C11	−1.5 (2)	C21—C20—C25—C24	−1.3 (2)
S1—C7—C12—C11	−179.05 (12)	S2—C20—C25—C24	175.38 (11)
C2—C1—N1—S1	−103.58 (13)	C15—C14—N2—S2	102.29 (14)
C6—C1—N1—S1	132.45 (12)	C19—C14—N2—S2	−133.55 (12)
C1—N1—S1—O1	175.08 (11)	C14—N2—S2—O4	−174.30 (12)
C1—N1—S1—O2	46.28 (13)	C14—N2—S2—O5	−45.13 (14)
C1—N1—S1—C7	−67.40 (13)	C14—N2—S2—C20	68.98 (14)
C8—C7—S1—O1	−95.15 (13)	C21—C20—S2—O4	134.13 (12)
C12—C7—S1—O1	82.45 (13)	C25—C20—S2—O4	−42.57 (13)
C8—C7—S1—O2	33.11 (14)	C21—C20—S2—O5	5.55 (14)
C12—C7—S1—O2	−149.29 (12)	C25—C20—S2—O5	−171.15 (11)
C8—C7—S1—N1	148.68 (12)	C21—C20—S2—N2	−110.23 (12)
C12—C7—S1—N1	−33.72 (14)	C25—C20—S2—N2	73.07 (13)

### Hydrogen-bond geometry (Å, °)

**Table:**

*D*—H···*A*	*D*—H	H···*A*	*D*···*A*	*D*—H···*A*
N1—H1N···O6^i^	0.83 (2)	2.00 (2)	2.8255 (17)	175.0 (18)
N2—H2N···O3^ii^	0.82 (2)	2.00 (2)	2.8155 (18)	173.1 (19)
O3—H3O···O5^iii^	0.83 (2)	1.93 (2)	2.7489 (15)	171 (2)
O6—H6O···O2^iv^	0.83 (2)	1.98 (2)	2.8001 (15)	169 (2)

Symmetry codes: (i) *x*−1, *y*−1, *z*; (ii) *x*+1, *y*+1, *z*; (iii) *x*, *y*−1, *z*; (iv) *x*, *y*+1, *z*.

## References

[bb1] Bergmeier, S. (2000). *Tetrahedron*, **56**, 2561–2576.

[bb2] Brandenburg, K. (1998). *DIAMOND* Crystal Impact GbR, Bonn, Germany.

[bb3] Bruker (2005). *APEX2* and *SAINT* Bruker AXS Inc., Madison, Wisconsin, USA.

[bb4] Chinnakali, K., Poornachandran, M., Raghunathan, R. & Fun, H.-K. (2007). *Acta Cryst.* E**63**, o1030–o1031.

[bb5] Coote, S. C., O’Brien, P. & Whitwood, A. C. (2008). *Org. Biomol. Chem.* **6**, 4299–4314. 10.1039/b811137e 19005588

[bb6] Farrugia, L. J. (1997). *J. Appl. Cryst.* **30**, 565.

[bb7] Krzemiński, M. P. & Wojtczak, A. (2005). *Tetrahedron Lett.* **46**, 8299–8302.

[bb8] Liu, Z., Fan, Y., Li, R., Zhou, B. & Wu, L. (2005). *Tetrahedron Lett.* **46**, 1023–1025.

[bb9] Naiker, T., Datye, A. & Friedrich, H. B. (2008). *Appl. Catal. A*, **350**, 96–102.

[bb10] Nan, Z.-H. & Xing, J.-D. (2006). *Acta Cryst.* E**62**, o1978–o1979.

[bb11] Sheldrick, G. M. (2008). *Acta Cryst.* A**64**, 112–122. 10.1107/S0108767307043930 18156677

